# Comparative Evaluation of the Increase in Enamel Hardness Post-External Bleaching after Using Casein Phosphopeptide Amorphous Calcium Phosphate Fluoride (CPP-ACPF) and 5% Sodium Fluoride (NaF) Remineralizing Agents

**DOI:** 10.1055/s-0043-1761189

**Published:** 2023-01-30

**Authors:** Irmaleny Irmaleny, Opik Taofik Hidayat, Yolanda Yolanda, Elisabeth Lumban Tobing

**Affiliations:** 1 Department of Conservative Dentistry, Faculty of Dentistry, Universitas Padjadjaran, Bandung, Indonesia; 2 Mandau General Public Hospital, Duri, Indonesia

**Keywords:** bleaching, enamel hardness, CPP-ACPF, 5% NaF, remineralization

## Abstract

**Objective** The purpose of this study was to analyze the increase in enamel hardness post-external bleaching after remineralized with casein phosphopeptide amorphous calcium phosphate fluoride (CPP-ACPF) and 5% sodium fluoride (NaF) and the difference in increased enamel hardness between CPP-ACPF and NaF materials.

**Materials and Methods** The study was true experimental *in vitro* using 30 samples of mandibular premolars. All samples were bleached using 40% hydrogen peroxide. The samples were divided into three groups of 10 samples each; group I as a control without application of remineralization material, group II was given an application of CPP-ACPF (GC Tooth Mousse Plus: GC Europe, Lot #201130B), and group III was given an application of 5% NaF (Clinpro White Varnish: 3M ESPE, Lot #NA62322) Then the entire samples were stored in artificial saliva. The hardness of the samples was measured using a Vickers hardness tester before bleaching, after bleaching, and after remineralization for 7, 14, and 21 days.

**Statistical Analysis** Analysis of the data used was an analysis of variance test to assess differences in the increase in enamel hardness between groups and paired *t* -tests and to determine differences in enamel hardness in each group.

**Results** This study showed that there was an increase in the enamel hardness after bleaching which was remineralized with CPP-ACPF and 5% NaF. There was a difference in the increased enamel hardness between teeth remineralized with CPP-ACPF and 5% NaF. The enamel hardness of CPP-ACPF was higher compared with 5% NaF after remineralization of 7, 14, and 21 days.

**Conclusion** There was an increase in the enamel hardness after external bleaching that was remineralized with CPP-ACPF and 5% sodium fluoride (NaF). There was a difference in the increased enamel hardness between teeth remineralized with CPP-ACPF and 5% sodium fluoride (NaF). CPP-ACPF showed a higher enamel hardness value than 5% NaF.

## Introduction

Bleaching is one of the cosmetic dentistry treatments that is often done because it is easy, efficient, and noninvasive to brighten discolored teeth. 1 2 Side effects of bleaching procedures such as tooth sensitivity, changes in enamel structure, and pulp cell damage are of significant concern among dentists. 3 Some studies have shown that bleaching can also soften the hard tissues of teeth, alter the morphology and mineral content of enamel, as well as a decrease the microhardness of enamel. ^1.2^

In-office bleaching techniques using high concentrations of hydrogen peroxide are effective in brightening teeth; however, this material can weaken the surface structure of enamel. 4 Some research results say that the use of high concentrations of hydrogen peroxide can lead to changes in mineral composition, morphological changes, and a decrease in the microhardness of enamel. 5 The application of remineralization materials either before or after bleaching can minimize demineralization that has an impact on enamel hardness. 6

Remineralization can either occur naturally or induced by remineralization agents that precipitate into the dental structure. 7 In the early stages of white spot lesion, remineralizing agents that contain 5% fluoride or casein phosphopeptide amorphous calcium phosphate fluoride (CPP-ACP) could be used as noninvasive treatment. 8 Fluoride is one of the remineralization materials that has proven effective in the prevention of dental caries. Fluoride will form fluorapatite crystals with the help of calcium and phosphate ions on the enamel surface that will inhibit demineralization and increase the microhardness value of enamel. 9 CPP-ACPF besides containing CPP-ACP also added 0.2% (900 ppm) sodium fluoride (NaF). CPP-ACPF applications showed a higher increase in enamel hardness. 10 NaF is one type of topical fluoride that is often used in dentistry. The application of fluoride will form a layer of calcium fluoride (CaF _2_ ) that then diffuses into the surface of the enamel to form fluorapatite. Thus, helping to increase remineralization which can increase the hardness of the enamel. 9

This study aimed to analyze whether there is an increase in enamel hardness post-external bleaching after remineralized with CPP-ACPF and 5% NaF and the difference in increased enamel hardness post-external bleaching between teeth remineralized with CPP-ACPF and 5% NaF. The research hypothesis is that there is no difference in post-bleaching enamel hardness remineralized with CPP-ACPF and 5% NaF.

## Materials and Methods

An *in vitro* study used 30 samples of mandibular premolars extracted based on the central limit theorem. The central limit theorem was a statistical theory where a sample was taken randomly from a population (30 samples or more); then the average value of the sample had a standard normal distribution. 11 The teeth were free of caries, fillings, erosion, and cracks, to obtain standard enamel surfaces. The crowns were separated from the roots 2 mm from the cementoenamel junction using a carborundum disc. The tooth fragments were positioned in an acrylic resin cylinder with the labial surface exposed. The enamel surfaces were ground and polished using #1200-grit silicon carbide paper.

The measurement of the enamel hardness value in the sample was performed using a Vickers hardness tester with a load of 200 g for 10 seconds at three points of the central labial surface of the tooth. The average value of the three points was used as the hardness value of the sample. All samples were bleached using 40% hydrogen peroxide (Opalescence Boost: Ultradent Product Inc., Lot #BL4SL) three times for 20 minutes. The samples were randomly divided into three groups consisting of 10 samples each, group I as a control without the application of remineralization material, group II was given the application of CPP-ACPF (GC Tooth Mousse Plus: GC Europe, Lot #201130B) 3 minutes twice a day for 21 days, and group III was given the application of 5% NaF (Clinpro White Varnish: 3M ESPE, Lot #NA62322) once and left for 4 hours. Then the entire sample is rinsed and stored in artificial saliva that is replaced daily. Sample hardness was measured before bleaching, after bleaching, and after remineralization at 7, 14, and 21 days.

## Results

Analysis of the data used was an analysis of variance (ANOVA) test to assess differences in the increase in enamel hardness between groups and paired *t* -tests to determine differences in enamel hardness in each group. Enamel hardness after bleaching treatment decreased significantly compared with before bleaching treatment in all three groups (control, CPP-ACPF, and 5% NaF). The results of enamel hardness testing before and after bleaching treatment can be seen in Table 1 .

**Table 1:** Results of the enamel hardness before and after bleaching

Treatment	n	Mean		SD	
		Before bleaching	After bleaching	Before bleaching	After bleaching
Control	10	411.10	333.00	11.350	25.924
CPP-ACPF	10	411.40	357.45	11.735	12.716
5% NaF	10	400.00	353.05	8.580	17.574
Total	30	407.50	347.83	11.605	21.720

Abbreviations: CPP-ACPF, casein phosphopeptide amorphous calcium phosphate fluoride; 5% NaF, 5% sodium fluoride; SD, standard deviation.

Enamel hardness has increased significantly after remineralization treatment. The results of the enamel hardness testing after 7,14, and 21 days of remineralization in the CPP-ACPF and 5% NaF group can be seen in Table 2 .

**Table 2:** Results of enamel hardness after remineralization treatment at 7, 14, and 21 days

Treatment	Days	Mean	*n*	SD
CPP-ACPF	7	415.45	10	9.335
	14	420.20	10	6.197
	21	422.00	10	5.715
5% NaF	7	413.05	10	7.448
	14	415.65	10	5.056
	21	421.65	10	6.334

Abbreviations: CPP-ACPF, casein phosphopeptide amorphous calcium phosphate fluoride; 5% NaF, 5% sodium fluoride; SD, standard deviation.

Furthermore, a follow-up test was performed using paired *t* -test to determine the difference in enamel hardness in the CPP-ACPF and 5% NaF groups after remineralization treatment for 7, 14, and 21 days. The results of the paired *t* -test analysis can be seen in Table 3 .

**Table 3:** Results of paired *t* -test analysis of enamel hardness after remineralization treatment at 7, 14, and 21 days

		5% NaF (7 days)	5% NaF (7 days)	5% NaF (7 days)	5% NaF (7 days)	5% NaF (7 days)	5% NaF (7 days)
		413.05	415.45	415.65	420.20	421.65	422.00
**5% NaF** **(7 days)**	413.05						
**CPP-ACPF** **(7 days)**	415.45	0.4350					
**5% NaF** **(14 days)**	415.65	0.3980	0.9480				
**CPP-ACPF** **(14 days)**	420.20	0.0228	0.1254	0.1418			
**5% NaF** **(21 days)**	421.65	0.0067	0.0471	0.0544	0.6366		
**CPP-ACPF** **(21 days)**	422.00	0.0049	0.0363	0.0422	0.5577	0.9091	

Abbreviations: CPP-ACPF, casein phosphopeptide amorphous calcium phosphate fluoride; 5% NaF, 5% sodium fluoride.

**Fig. 1:**
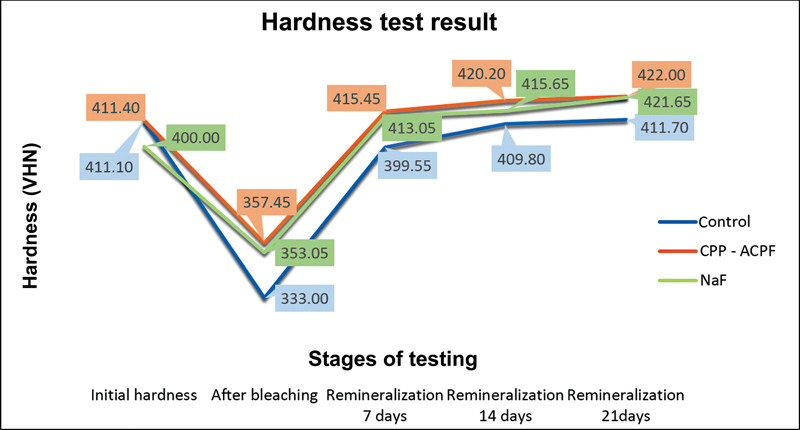
Diagram of differences in increased enamel hardness after remineralization treatment in control, casein phosphopeptide amorphous calcium phosphate fluoride (CPP-ACPF), and 5% sodium fluoride (5% NaF) groups. The results of the study showed that the increase in post-bleaching enamel hardness in the CPP-ACPF group was higher than the 5% NaF group, although there was no statistically significant difference.

The difference in the increase of enamel hardness between the control group and the remineralized group with CPP-ACPF and 5% NaF can be seen in Fig. 1 .

## Discussion

Based on the results of the analysis using the ANOVA test, there was a significant decrease in the enamel hardness value after bleaching with 40% hydrogen peroxide compared with the initial hardness. This decrease in hardness was caused by changes in mineral content in the form of demineralization in the enamel structure. The enamel demineralization process after bleaching is related to the composition and concentration of the bleaching material, the contact time and the method of application of the bleaching material to the teeth, the pH value, and the type of solution in the bleaching material. 12 13

Hydroxyapatite crystals are the components that makeup enamel hardness composed of the main inorganic matrix in the form of calcium and phosphate. 14 Bleaching treatment with high concentrations of hydrogen peroxide detaches calcium and phosphate ions of the enamel surface, thereby reducing the microhardness of the enamel. Mondelli et al research on the side effects of hydrogen peroxide bleaching material on enamel shows that the higher the concentration of bleaching material, the higher the demineralization of enamel that occurs. 15 Oxidation–reduction reactions of bleaching materials produce hydrogen ions (H ^+^ ) that can create an acidic environment and cause the dissolution of organic and inorganic enamel matrices. 16

Remineralization using fluoride after bleaching has been shown to restore the microhardness of the enamel surface. Fluoride compounds can restore damaged tooth microstructure through the absorption and sedimentation of salivary components such as calcium and phosphates. 17 The formed calcium fluoride (CaF _2_ ) layer deposited on the surface of the enamel crystals forming fluorapatite. 18

In the control group, the enamel hardness did not increase compared with the initial hardness after 7 days in artificial saliva, but on day 21 it returned to the initial hardness. Saliva plays a role in returning calcium and phosphate ions to the demineralized enamel surface, thereby increasing remineralization. 19 Although artificial saliva was used in this study, it was not able to restore the initial enamel hardness in a short time due to the limited amount of minerals. Remineralization materials are still needed to prevent side effects of post-bleaching demineralization such as sensitivity, changes in morphological structure, and enamel hardness. ^16.19^

In the CPP-ACPF group, the enamel hardness increased beyond the initial hardness after CPP-ACPF application for 7 days, then increased further on day 14 and experienced a slight increase on day 21. CPP-ACPF contains calcium, phosphate ions, and 0.2% (900 ppm) NaF. CPP are able to stabilize calcium, phosphate, and fluoride ions in nanocomplexes of ACPF solutions. 16 CPP-ACPF acts as a reservoir of calcium and phosphate ions available in natural saliva and maintains the solution in a supersaturation state, thereby increasing the remineralization and enamel crystal hardness. 20 Aras et al research found that CPP-ACPF remineralization was effective in increasing enamel surface hardness in artificial enamel caries. 21 Llena et al and Heshmat et al research showed that the use of CPP-ACPF for 2 weeks post-bleaching can increase remineralization and restore enamel hardness. ^16.22^

In the 5% NaF (3M Clinpro White Varnish) group, the enamel hardness increased beyond the initial hardness after 5% NaF application for 7 days, slightly increased at 14 days, and further increased at 21 days. Clinpro White Varnish contains tricalcium phosphate and 5% (22,600 ppm) NaF. 22 Dionysopoulos et al concluded that topical application of fluoride during and after bleaching treatment significantly increased the surface microhardness of the enamel. 23 Topical application of high concentrations of fluoride will form a layer of calcium fluoride (CaF _2_ ) on the enamel surface to inhibit demineralization or decrease in enamel hardness. 24

## Conclusion

Based on the results of the study, it can be concluded that there was an increase in the enamel hardness after external bleaching that was remineralized with CPP-ACPF and 5% NaF. There was a difference in the increased enamel hardness between teeth remineralized with CPP-ACPF and 5% NaF. CPP-ACPF showed a higher enamel hardness value than 5% NaF. The limitation of this study was used Vickers hardness tester only. Further experimental research is needed to determine the remineralization of enamel hardness.

From the results of this study, it is suggested that patients should be given the application of a remineralizing agent using CPP-ACPF or 5% NaF after bleaching treatment.
